# Tautomerism of 4,4^′^-dihydroxy-1,1^′^-naphthaldazine studied by experimental and theoretical methods

**DOI:** 10.1186/1752-153X-7-29

**Published:** 2013-02-11

**Authors:** Anife Ahmedova, Svilen P Simeonov, Vanya B Kurteva, Liudmil Antonov

**Affiliations:** 1 Faculty of Chemistry and Pharmacy, Sofia University “St. Kliment Ohridski”, 1, J. Bourchier blvd., Sofia, 1164, Bulgaria; 2 Institute of Organic Chemistry with Centre of Phytochemistry Bulgarian Academy of Sciences, Acad. G. Bonchev str.; bl.9, Sofia, BG-1113, Bulgaria

**Keywords:** 4,4^′^-dihydroxy-1,1^′^-naphthaldazine, Flash photolysis, Quantum chemical calculations, Tautomerism, Tautomeric constants

## Abstract

**Background:**

The title compound belongs to the class of bis-azomethine pigments. On the basis of comparative studies on similar structures, insight into the complex excited state dynamics of such compounds has been gained. It has been shown, for example, that only compounds that possess hydroxyl groups are fluorescent, and that the possibility for *cis-trans* isomerisation and/or bending motions of the central bis-azomethine fragment allows for different non-radiative decay pathways.

**Results:**

The compound, 4,4'-dihydroxy-1,1'-naphthaldazine (1) was synthesized and characterized by means of spectroscopic and quantum chemical methods. The tautomerism of 1 was studied in details by steady state UV-Vis spectroscopy and time resolved flash photolysis. The composite shape of the absorption bands was computationally resolved into individual subbands. Thus, the molar fraction of each component and the corresponding tautomeric constants were estimated from the temperature dependent spectra in ethanol.

**Conclusions:**

According to the spectroscopic data the prevalent tautomer is the diol form, which is in agreement with the theoretical (HF and DFT) predictions. The experimental data show, however, that all three tautomers coexist in solution even at room temperature. Relevant theoretical results were obtained after taking into account the solvent effect by the so-called supermolecule-PCM approach. The TD-DFT B3LYP/6-31 G** calculated excitation energies confirm the assignment of the individual bands obtained from the derivative spectroscopy.

## Introduction

**Figure 1:**
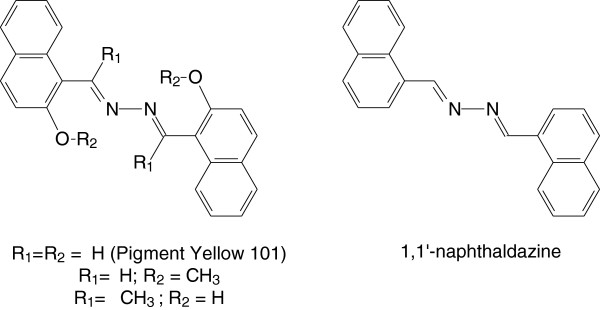
**Molecular structure of Pigment Yellow 101 and other derivatives of 1,1**^**′**^**-naphthaldazine.**

The studied compound, 4,4^′^-dihydroxy-1,1^′^-naphthaldazine (1), belongs to the class of bis-azomethine pigments. The most studied example is the Pigment Yellow 101 (P.Y.101), depicted in Figure 1, which is the only commercially available fluorescent yellow pigment that shows strong fluorescence in crystalline state. The fascinating photochemical properties of P.Y. 101 have been subject of intense studies combining crystallography, time resolved spectroscopy [1,2] and high-level quantum chemical calculations [3-5]. Based on comparative studies on similar structures, shown in Figure 1, it has been possible to gain insight into the complex excited state dynamics of these compounds. It has been shown that only the compounds that possess hydroxyl groups are fluorescent. The presence of intramolecular hydrogen bonding has been proved to be responsible for the ultrafast ESIPT (excited-state intramolecular proton transfer) process and also for the high photostability of P.Y.101. Additional intramolecular rearrangements, such as *cis-trans* isomerisation and/or bending motions of the central bis-azomethine fragment, play also important role in the different non-radiative decay pathways.

**Figure 2:**
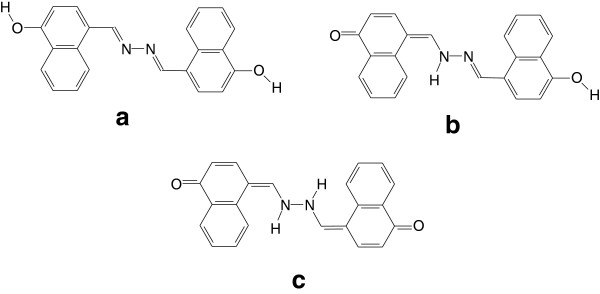
Schematic representation of possible tautomers of compound 1.

Accordingly, we focused our study on a compound that possesses hydroxyl group but no intramolecular hydrogen bonding is present i.e. 4,4^′^-dihydroxy-1,1^′^-naphthaldazine (1). The structure of the title compound is given in Figure 2 together with the possible tautomeric forms. The spectral properties of compound 1 were studied by means of steady state electron spectroscopy accounting for various external factors, such as temperature, solvent polarity and acidity of the medium. Additionally, laser flash photolysis was used to get an idea about the proton exchange under laser excitation. The structure and the spectroscopic properties of the possible isomers of 1 were evaluated by means of quantum chemical calculations in order to find plausible explanation of the experimentally observed spectroscopic data.

## Experimental

### Synthesis and spectroscopic measurements

All solvents and reagents used were AR grade. Fluka silica gel/TLC-cards 60778 with fluorescent indicator 254 nm were used for TLC chromatography and R_f_-value determination. The melting point was determined in capillary tube on MEL-TEMP 1102D-230 VAC (Dubuque, IA, USA) apparatus without corrections.

The title compound, N,N’-bis-(4-hydroxy-[1]naphthylmethylene)-hydrazine (1), was obtained from naphthaldehyde according to a standard procedure; i.e. to a solution of 1-hydroxy-4-naphthaldehyde (1 mmol) in EtOH (2 ml) hydrazine hydrate (0.5 mmol) was added and the mixture was stirred at room temperature for 4 h. No acid catalyst was used in an attempt to avoid the protonation of the product. The residue formed was filtered off, washed with EtOH and then with Et_2_O, and dried in air to give 60% yield of 1 as a yellow powder; m. p. 235-236 °C (236°C)[6]; R_f_ - 0.15 (EtOAc:hexane 2:1); ^1^H NMR 7.03 (d, 2H, J 8.0, C*H*-2 Ar), 7.58 (ddd, 2H, J 1.1, 6.8, 8.2, C*H*-7 Ar), 7.70 (ddd, 2H, J 1.5, 6.8, 8.4, C*H*-8 Ar), 7. 97 (d, 2H, J 8.2, C*H*-3 Ar), 8.30 (dd, 2H, J 1.1, 8.2, C*H*-6 Ar), 9.25 (s, 2H, C*H*=N), 9.30 (d, 2H, J 8.4, C*H*-9 Ar), 10.96 (bs, O*H*) ppm; ^13^C NMR 108.5 (*C*H-2 Ar), 120.9 (*C*_quat_-4), 123.1 (*C*H-6 Ar), 125.2 (*C*_quat_-10), 125.6 (*C*H-9 Ar), 125.8 (*C*H-7 Ar), 128.3 (*C*H-8 Ar), 132.7 (*C*_quat_-5), 133.4 (*C*H-3 Ar), 157.2 (*C*_quat_-1), 162.0 (*C*H=N) ppm; COSY cross peaks 7.03/7.97, 7.58/7.70, 7.58/8.30, 7.70/9.30; HSQC cross peaks 7.03/108.5, 7.58/125.8, 7.70/128.3, 7.97/133.4, 8.30/123.1, 9.25/162.0, 9.30/125.6.

The IR spectrum was recorded on a Bruker IFS-113 FTIR Spectrometer in KBr. The NMR spectra were recorded on a Bruker Avance DRX 250 (for 1D) and Bruker Avance II+ 600 (for 2D) spectrometers in DMSO-d_6_. The chemical shifts were quoted in ppm in δ-values against tetramethylsilane (TMS) as an internal standard and the coupling constants were calculated in Hz. The assignment of the signals in 1D NMR spectra is based on the observed cross peaks in 2D homo- and heteronuclear correlations COSY and HSQC, respectively. The UV-Vis spectral measurements were performed on a JASCO V-570 UV-Vis-NIR spectrophotometer, equipped with a Julabo ED5 thermostat (precision 1°C), in spectral grade solvents. The obtained spectral curves were processed by a software for overlapping bands decomposition [7,8] and for derivative spectroscopy, developed by us [9]. Laser flash photolysis experiments were performed using a setup that has been described previously [10]. Solutions were placed in quartz cells (4.5 cm long and 1 cm wide) and excited by one of the following excitation sources: a Lambda-Physik EMG 101 excimer laser operating at 308 nm (XeCl) with a pulse energies of *ca.* 100 mJ and pulse widths of *ca.* 30 ns. The photochemical stability of the samples was monitored.

### Quantum chemical calculations

The quantum chemical calculations were performed with full geometry optimization without any symmetry restrictions using the Gaussian 03 and Gaussian 09 program packages [11]. In order to evaluate the ground state properties of the studied compound, its potential energy surface was searched for stable conformers. Geometries of nineteen possible rotamers and tautomers were optimized by the semiempirical AM1, *ab initio* Hartree-Fock and DFT methods. Vibrational frequencies were computed in order to verify that local energy minima were attained. Selected structures were additionally optimized using the density functional theory (DFT) and two hybrid B3LYP and M06-2X functionals [12-14] with two different basis sets, 6-31G** and def2TZVP [15]. Vertical excitation energies were calculated employing ZINDO and time-dependent DFT (TD-DFT) with the B3LYP/6-31G** at the equilibrium geometries of the most stable conformers. The solvent effect was taken into consideration by the polarizable continuum model [16,17] and IEF-PCM/B3LYP geometry optimization at the 6-31G** level in methanol were carried out using the standard united-atom cavity model, as implemented in G03 software. Solvent-solute interactions were modeled adding one methanol molecule close to the enol OH group of compound 1 so that intermolecular hydrogen bond, of type (solv)O….H-O, is formed. The models were optimized by B3LYP/6-31G** method, and the supermolecule-PCM approach was employed, doing IEF-PCM/B3LYP geometry optimization at the 6-31G** level in methanol of the optimized solute-solvent complexes, similarly to some azonaphthols [18,19].

## Results and discussion

### Spectroscopic data

The optical properties of compound 1 were studied by means of steady state absorption and emission spectroscopy and laser flash photolysis. Fluorescence measurements of ethanol solution of 1 showed that it is not fluorescent. Comparing with the fluorescent properties of the hydroxyl-group containing P.Y.101 and its derivatives, it can be concluded that the presence of intramolecular hydrogen bonding is operative for their strong fluorescence, while the competitive isomerisation *via* bond rotation might serve as a non-radiative decay pathway. This is in line with the observation of very strong fluorescence of P.Y.101 in solid state [1].

**Figure 3:**
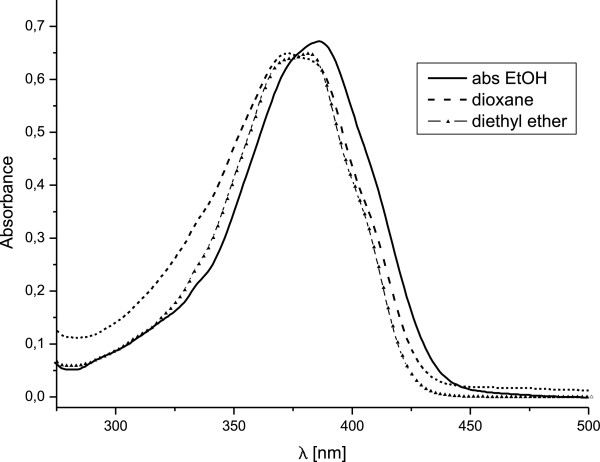
Normalized spectra of compound 1 in absolute ethanol, diethyl ether and dioxane.

Absorption spectra of compound 1 were recorded in various solvents and the normalized spectra in absolute ethanol, diethyl ether and dioxane are presented in Figure 3. Bathochromic shift is observed going from diethyl ether to ethanol and DMSO showing maxima in the spectra at 381, 386 and 393 nm, respectively. Although that there is no significant difference in the shape of the absorption band (complex contour containing number of individual subbands, see Figure 3), we will emphasize on the change in relative intensities of the subbands, composing the overall contour, in three of the solvents. The observed shift in the position of the absorption maximum in these solvents is most probably caused by redistribution of the intensities of the individual subbands. In addition, the intensity of the shoulder at *ca.* 330 nm decreases when going from diethyl ether to ethanol. Complete list of the apparent maxima in ten different solvents and the estimated position of the subbands, obtained from derivative spectroscopy, is presented in Additional file 1: Table S1. Taking into account the possible tautomerism in this compound, the observed spectral changes can be attributed to solvent caused shift in the position of the tautomeric equilibrium [20].

**Figure 4:**
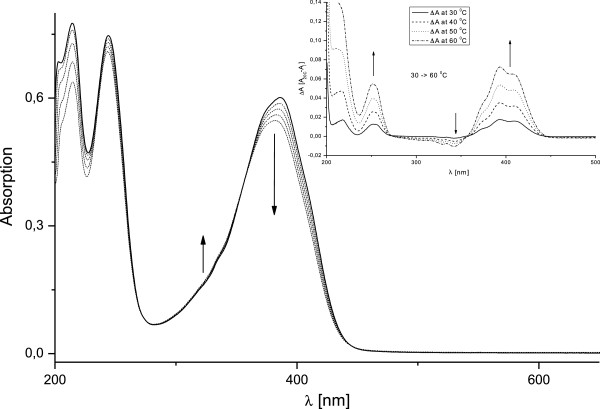
**Temperature dependence of the absorption spectra of 1 in absolute ethanol (1.7** × **10**^**-5**^ **M), recorded in the temperature range 20 – 60°C. Solid line - 20°C, dashed lines correspond to 30, 40, 50 and 60°C.** In the inset the difference spectra are shown.

For further confirmation of the presence of tautomeric equilibrium in solution, temperature dependent absorption spectra were recorded in three different solvents - ethanol, DMSO and chloroform. The spectra in ethanol, recorded in the temperature range 20 – 60°C, are presented in Figure 4. Virtually the same temperature-dependent spectra were obtained in DMSO and chloroform. In all three solvents the spectral changes upon temperature elevation are characterized by a slight increase at *ca*. 330 nm and a decrease of the maximum at *ca*. 390 nm (see the difference spectra in the inset of Figure 4). Although the observed changes in the spectral shape and the related isosbestic points are not very well pronounced, they give the first indication that at least two species coexist in solution. Initially, the species absorbing at 386 and 330 nm were referred to as A and B, respectively, and the corresponding equilibrium constant, K_T_=[B]/[A], was estimated from the temperature dependent spectra in ethanol. Accordingly, the calculated values for ΔG are in the range of 2.26 to 1.53 kcal/mol (without accounting for the solvent contraction) and the corresponding values for ΔH and ΔS are estimated to 9.48 kcal/mol and - 24.01 cal/mol.K, respectively (Additional file 2: Table S2). Considering the complexity of the shape of the absorption band, we sought for additional experimental evidences for coexistence of tautomers in solution, as well as defining their types. Therefore, decomposition procedure and derivative spectroscopy were employed for the absorption spectra, and flashphotolysis experiments were performed.

**Scheme 1:**
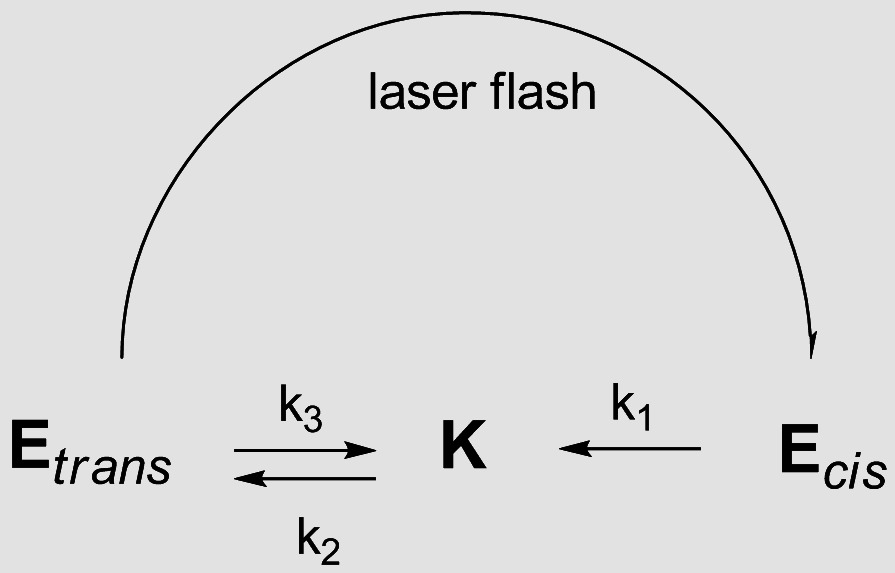
Possible tautomerization processes of compound 1 and the corresponding rate constants.

The different electronic structure of the tautomers, containing enol and keto fragment, determines quite different photochemical behavior. According to previous flash photolysis study of azonaphthol tautomeric compound 1-phenylazo-4-naphthol [21] upon absorption of a photon the E-form undergoes *trans*-*cis* isomerization (Scheme 1), whereas the keto tautomer does not show any detectable transient signal. In addition, the *cis* enol tautomer does not obey the direct *cis*-*trans* relaxation, being initially converted to the K-form and then to the corresponding enol form in order to restore the initial equilibrium tautomeric ratio. The process of relaxation of E_*cis*_ to K is governed by a rate constant k_1_ and the restoration of the tautomeric equilibrium is defined by k_II_ = k_2_ + k_3_.

**Figure 5:**
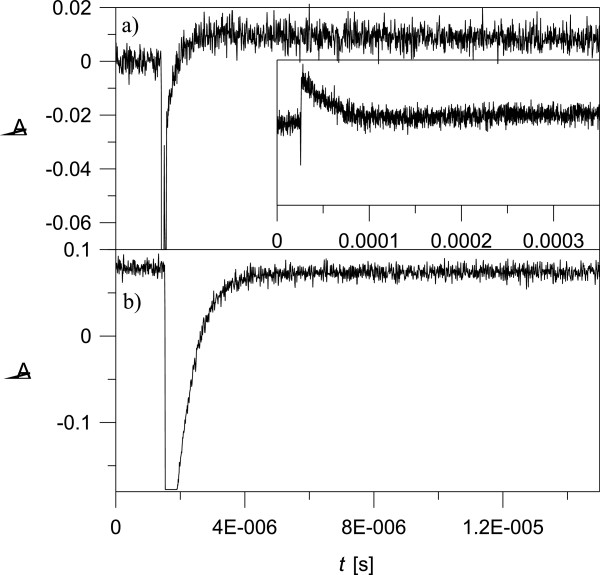
Time dependence of the transient absorption of 1 in methanol solution at 340 nm (a) and 400 nm (b).

The kinetic curves for solution of compound 1 in methanol, measured at 340 and at 400 nm, presented in Figure 5, show a typical keto and enol behavior, respectively. As can be seen from Figure 5 a, at 340 nm, there is a fast process (k_I_=3.18 ± 0.22 × 10^6^ s^-1^) of accumulation of the keto tautomer, followed by a relatively slow process (k_II_=3.61 ± 0.13 × 10^4^ s^-1^) of returning to the equilibrium state (Figure 5 a inset). The changes at 400 nm can be attributed to the initial decrease of the *trans* enol form, converted by the laser flash to *cis* form, followed by a rise - restoration of the equilibrium state through the keto form. The process is complicated and consists of fast and slow components with rate constants similar to those measured at 340 nm. This behavior can be attributed to the complexity of the tautomers in 1, where except the pure keto (c) and enol (a) tautomers, a mixed keto-enol form exists (b).

Based on the results from flash photolysis experiments it can be concluded that the species absorbing at 330 nm is the diketo tautomer (in Figure 2c) of compound 1 and coexists with the most stable diol tautomer (in Figure 2a). It might be expected, however, that the monoketo tautomer (in Figure 2b), being intermediate between (a) and (c), also exists in the solution and this was actually suggested from the flash photolysis kinetic curves. In search of further confirmation to this supposition we performed a detailed assessment of the composite shape of the absorption spectrum observed in ethanol. A mathematical curve decomposition procedure for quantitative analyses of tautomeric equilibria, developed by some of us, [7] was applied for the temperature dependent spectra of compound 1 in ethanol.

The temperature dependent spectra in ethanol were decomposed into 7 components, which were further attributed to the three possible tautomers of compound 1. The latter was done by monitoring the change in integral area of each component with temperature increase. Additionally, second derivative spectra were used to verify the position of the components’ absorption maxima. Three of the components with absorption maxima at 410, 380 and 354 nm show gradual decrease of the calculated area with temperature increase and were attributed to the most abundant diol form (a). The sum of the integral areas of these three components is denoted as *I*^*i*^_*diol*_. The other two components exhibit maxima at 392 and 324 nm and are attributed to the monoketo (b) and the diketo (c) forms, respectively, with the corresponding integral areas *I*^*i*^_*monoketo*_ and *I*^*i*^_*diketo*_. The data were used to calculate the molar fractions of each component using equation (1) (for details see Additional file 3)

(1) $$\frac{I_{\text{diketo}}^{i}}{I_{\text{diketo}}^{0}}+\frac{I_{\text{monoketo}}^{i}}{I_{\text{monoketo}}^{0}}+\frac{I_{\text{diol}}^{i}}{I_{\text{diol}}^{0}}=1$$

As can be seen from Table 1, the diol form (a) is the main component at 20°C. The quantity of the diketo form (c) exceeds the monoketo tautomer (b). With the temperature increase the quantity of diol form diminish, whereas those of the other two species rise gradually. At 60°C the quantity of the diketo form prevails that of the diol form, while the presence of the monoketo tautomer is *ca.* 10%. The current observation is in accordance with the temperature dependent tautomerism in 4-phenylazo-1-naphthol,[22] which shows lowering the quantity of the enol form by increasing the temperature in ethanol, whereas the opposite trend is observed in the 2-phenylazo-1-naphthol, 1-phenylazo-2-naphthol and the corresponding Schiff bases. In the case of compound 1 the enol – keto tautomerism takes place in two steps a ↔ b ↔ c, which are referred to as process 1 and 2, respectively (in Table 1). From the calculated differences in the Gibbs free energies for these processes the enthalpy and the entropy were calculated, too. The obtained results are as follows: for process 1) ΔH_1_ = 5.66 ± 0.46 kcal/mol, ΔS_1_ = -13.76 ± 1.46 cal/mol.K; for process 2) ΔH_2_ = -4.17 ± 0.38 kcal/mol, ΔS_2_ = 9.11 ± 1.22 cal/mol.K.

**Table 1:** Calculated molar fractions of the components present in ethanol solution of 1 along with the corresponding equilibrium constants and the Gibbs free energy differences

**T [K]**	$X_{\mathrm{diol}}^{i}=\frac{I_{\mathrm{diol}}^{i}}{I_{\mathrm{diol}}^{0}}$ **a**	$X_{\mathrm{monoketo}}^{i}=\frac{I_{\mathrm{monoketo}}^{i}}{I_{\mathrm{monoketo}}^{0}}$ **b**	$X_{\mathrm{diketo}}^{i}=\frac{I_{\mathrm{diketo}}^{i}}{I_{\mathrm{diketo}}^{0}}$ **c**	$K_{T_{1}}^{i}=\frac{X_{\mathrm{monoketo}}^{i}}{X_{\mathrm{diol}}^{i}}$	$K_{T_{2}}^{i}=\frac{X_{\mathrm{diketo}}^{i}}{X_{\mathrm{monoketo}}^{i}}$	**ΔG**_**1 **_**[kcal/mol]**	**ΔG**_**2 **_**[kcal/mol]**
293	0.535	0.035	0.432	0.065	12.329	1.59	−1.46
303	0.519	0.039	0.438	0.075	11.214	1.56	−1.45
313	0.489	0.053	0.461	0.109	8.673	1.38	−1.34
323	0.463	0.069	0.468	0.148	6.813	1.22	−1.23
333	0.438	0.088	0.474	0.200	5.421	1.06	−1.12

**Figure 6:**
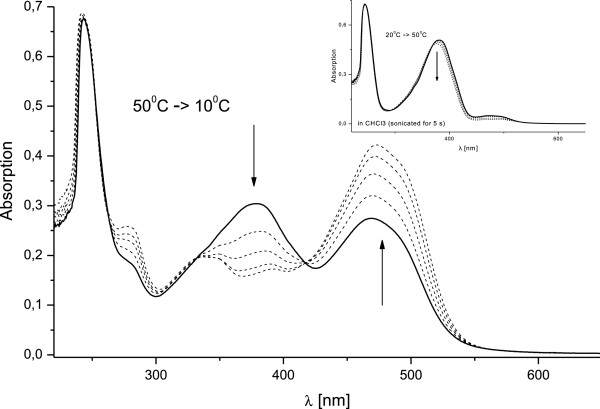
**Effect of temperature and sonication on the absorption spectra of chloroform solution of compound 1 (1.7** × **10**^**-5**^ **M) – temperature range 50 – 10°C, sonication time - 3 minutes; solid line - 50°C, the dashed lines correspond to 40, 30, 20 and 10°C.** The inset shows the temperature dependence (20 – 50°C) of a solution initially sonicated for 3 seconds.

Additionally to the observed spectral changes upon increasing the temperature, another phenomenon was observed in chloroform solutions. Due to the poorer solubility in chloroform the solutions were sonicated for certain time. As a result, spectral changes were detected corresponding to protonation of compound 1. The observed protonation is produced by the hydrochloric acid that is formed during the chloroform sonication through radical intermediates. The mechanism of the sonodegradation of trihalomethanes is well studied [23] and applications of the protonation of some compounds as sonochemical dosimeters are suggested [24]. In Figure 6 the spectra of compound 1 in chloroform are presented. The inset shows the temperature dependent spectra of a solution that was sonicated for 3 seconds. The observed temperature dependence is analogous to the one already described. However, additional sonication for 3 minutes at 50°C leads to the spectrum presented in Figure 6 with solid line. It can be clearly seen that a new intense band appears at 469 nm as a result from protonation of compound 1. Furthermore, the spectral changes upon decreasing the temperature down to 10°C were monitored in order to estimate the thermodynamic parameters of the observed equilibrium.

**Figure 7:**
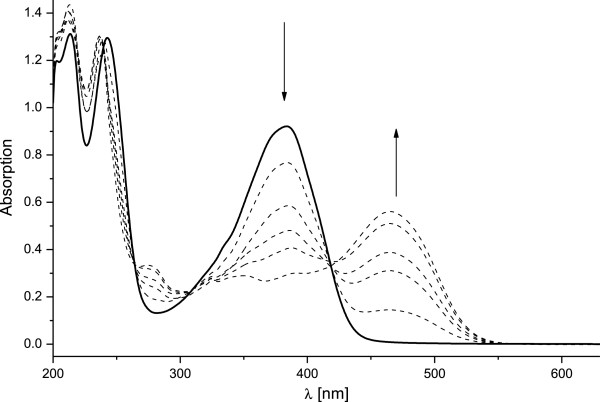
**Absorption spectra of 1 in methanol (2.7** × **10**^**-5**^ **M) with addition of HCl.** The solid line shows the spectrum without acid, dashed lines correspond to the spectra with increasing concentration of the acid added: 8 × 10^-6^ M; 1.6 × 10^-5^ M; 2.4 × 10^-5^ M; 3.6 × 10^-5^ M; 6.4 × 10^-5^ M.

In order to confirm these results, a series of spectra were recorded in methanol with increasing amount of added hydrochloric acid and are presented in Figure 7. The observed changes in the spectral shape are the same as the ones resulting from the sonication.

### Quantum chemical calculations

**Figure 8:**
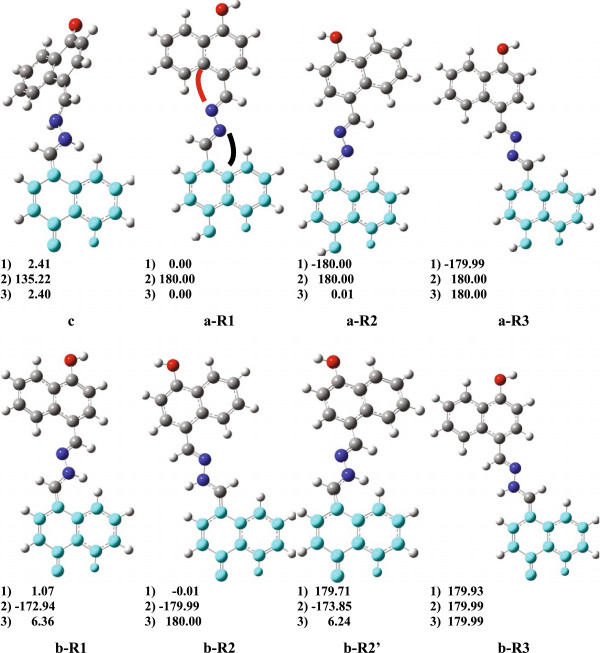
Optimized structures (B3LYP/6-31 G**) of the possible tautomers of compound 1 , diol (a), keto (b) with some of their isomers, and the diketo tautomer (c). The dihedral angles 1) and 3) are defined between the N- and quaternary C-atom, as shown in a-R1, angle 2) is the C-N-N-C, and their values are given. The lower, light-blue part of each molecule is oriented in the same way in all structures for better visualisation of the conformational differences between the calculated isomers.

The nineteen optimized conformers of 1 stem from the three possible tautomeric forms shown in Figure 2; the diol form (a) the keto form (b) and the diketo form (c). Moreover, the rotation around the N-N bond, leading to *cis* and *trans* isomers, was also taken into account. The *trans* isomers were found to be the preferred ones for all three tautomers. Furthermore, the rotation about the C-C bonds bearing the naphthyl rings leads to formation of different *endo* and *exo* isomers. All tautomers shown in Figure 2, are in *endo*-*endo* form (both N-atoms’ electronic lone pairs are directed towards the neighboring naphthalene rings). The DFT (B3LYP/6-31 G**) calculated energies of the most stable conformers of the tautomers a – c are listed in Table 2 and the optimized structures of the possible conformers of the tautomers (a), (b) and (c) are depicted in Figure 8. It must be noted that the most stable isomer of compound 1 is the *endo*-*endo* rotamer of the diol tautomer, a-R1 in Figure 8, whereas the crystal structure of P.Y.101 shows that it exist in the *exo*-*exo* form of the diol tautomer (similarly to the a-R3 in Figure 8). Apparently, the specific intramolecular interactions are operative for stabilization of the corresponding rotamers; the presence of intramolecular H-bond (N….H-O) in P.Y.101 stabilizes the *exo*-*exo* form, while in compound 1 the *endo*-*endo* rotamer is stabilized by the N….H-C interaction. The N….H-C distance in the optimized structure of a-R1 is 2.213 Å, its geometry is completely planar and it is the isomer with the lowest energy. Rotation about each C-C bond in the structure of the diol tautomers leads to the other two conformers of the diol tautomer - the a-R2 and a-R3 structures, which are also planar as could be seen from Figure 8. On the other hand, upon tautomerization the *endo*-*endo* rotamer is no longer the most stable one of the monoketo tautomer (b) but the *endo*-*exo* form b-R2 (see Figure 8 and Table 2). These data show that tautomerism of compound 1 is accompanied with rotation about the C-C bonds. As could be expected from streric point of view, the b-R2 form has also planar structure whereas the other two rotamers of the monoketo tautomer are slightly twisted (b-R1 and b-R2’). On the contrary, the optimized structures of the diketo tautomer (c) show almost perpendicular orientation of the naphthyl rings and it is hard to distinguish the different rotamers. Therefore, only the energy of the most stable form is given in Table 2 and in Figure 8.

**Table 2:** Calculated relative energies (in kcal/mol) and dipole moments for the most stable conformers of the possible tautomeric forms of compound 1 at the B3LYP/6-31 G** level (zero-point vibration energy correction included)

**Tautomer**	**Relative energies [kcal/mol]**	**Dipole moment [Debye]**
**a-R1**	**0.00**	0.00
**a-R2**	0.70	2.51
**a-R3**	1.43	0.00
**b-R1**	6.70	9.47
**b-R2**	**6.08**	9.30
**b-R2**^′^	7.21	8.69
**b-R3**	6.58	8.74
**c**	**21.78**	0.38

The optimized structures explain well the obtained dipole moments (Table 2) and the calculated vertical excitation energies (Table 3). While in tautomers (a) and (b), the relative planarity permits the conjugation between the electron donor and acceptor parts of the molecule, the structure of tautomer (c) clearly shows that such conjugation is not possible. Correspondingly, the TD-DFT calculated excitation energy for (c) is *ca.* 346 nm, while those for tautomers (a) and (b) are 367 and 393 nm, respectively. We shall emphasize that this results agree well with the assignment of the experimentally observed spectra based on the decomposition procedure and the derivative spectroscopy (*vide supra*). Unlike the calculated spectral properties, the theoretically predicted energies and relative stabilities in gas phase agree only qualitatively with the experimental observations. Therefore, we performed additional calculations using a fitted hybrid meta-GGA functional with 54% HF exchange - M06-2X, specially developed to describe main-group thermochemistry and non-covalent interactions, in combination with a large basis set, def2TZVP. The latter basis set was used also in calculation with the B3LYP functional and Hartree-Fock method. The *ab initio* HF calculations were perfomed for the sake of comparison, since it has been shown that the DFT methods do not predict correctly the relative energies of substituted azonaphthols [25]. All data from our calculations are summarized in tables given in Additional file 4. The main conclusions from the isolated-molecule gas-phase calculations are that both hybrid functionals with both basis sets give virtually the same geometrical and energetical picture, predicting too large energy differences between the different tautomers of compound 1 (up to 20 kcal/mol). Such a disagreement with the experiment could point out that the role of the media is not negligible and the specific interactions between the solute and the solvent molecules most possibly contribute by a large extent to the stabilization of the various tautomeric forms.

**Table 3:** Calculated vertical excitation energies (in nm) of the possible tautomers (a - c), their singly protonated forms* (a+, a+O), and bound methanol molecule to the corresponding tautomers (a_MeOH, b_MeOH) obtained by the ZINDO and TD-DFT –B3LYP/6-31 G** methods

**name**	**TDDFT**	**ZINDO**
**a**	368.83 nm (f=1.01)	354.18 nm (f=1.21)
**b**	393.30 nm (f=1.15)	379.22 nm (f=1.41)
**c**	346.46 nm (f=0.15)	344.28 nm (f=1.49)
	319.99 nm (f=0.80)	257.12 nm (f=0.60)
**a +**	528.25 nm (f=0.50)	454.06 nm (f=1.05)
	373.62 nm (f=0.56)	
**a+O**	575.49 nm (f=0.34)	404.56 nm (f=0.85)
	349.21 nm (f=0.48)	
**a_MeOH**	373.95 nm (f=0.98)	355.95 nm (f=1.22)
**b_MeOH**	397.34 nm (f=1.16)	381.71 nm (f=1.43)

Oscillator strength is given in parentheses.

*sites for protonation of species a+ and a+O are the N- and the O-atoms, respectively.

In order to account for the interaction between the solvent molecules and the solute, different types of adducts of compound 1 with a methanol molecule were modeled and optimized. Amongst them the most stable ones, for the corresponding tautomeric form, were those in which the methanol molecule is attached by intermolecular H-bonding to the OH-group of the compound 1. The energy difference between the diol (a) and the monoketo (b) forms, with attached methanol molecule, is 6.09 kcal/mol as obtained from the B3LYP/6-31 G** calculations. Furthermore, we reoptimized these solute-solvent complexes in methanol media using IEF-PCM calculation. The result from this supermolecule-PCM calculation lead to decrease of the energy difference between the diol (a) and the monoketo (b) forms down to 2.46 kcal/mol. The obtained relative energies from the PCM calculation of individual molecules and the supermolecule-PCM, including a methanol molecule, are compared in Table 4.

**Table 4:** B3LYP/6-31 G** calculated energy differences (in kcal/mol) between the diol form a-R1 and the monoketo form b-R3 of compound 1, compared with the IEF-PCM (solvent methanol) and the supermolecule-PCM calculations

**Tautomer**	**Relative energies [kcal/mol]**
**(a-b)**	6.58
**(a-b)× MeOH**	6.09
**(a-b) PCM**	2.40
**(a-b)×MeOH PCM**	2.46

As can be seen from Table 4, formation of intermolecular complex of (a) and (b) tautomers of compound 1 with a methanol molecule do not cause appreciable change in the corresponding energy difference. It is the polarizable continuum model that leads to a lower energy difference between the tautomers. Taking into account the calculated dipole moments of these tautomers it is reasonable that the more polar (b) tautomer is better stabilized in the polar methanol media. However, such effect cannot be expected for the relatively non-polar diketo tautomer (c). Indeed, the energy difference between (a) and (c) forms is lowered from 21.78 to 14.03 kcal/mol only, by inclusion the IEF-PCM in the DFT calculations. Nevertheless, this result does not agree quantitatively with the experimental data.

## Conclusion

The tautomerism in 4,4^′^-dihydroxy-1,1^′^-naphthaldazine (1) was studied by time resolved and steady state absorption spectroscopy. Temperature dependent spectra and laser flash photolysis indicate that the three possible tautomers are present in solution. The absorption spectra were decomposed into individual subbands in order to estimate the relative abundance of all species present in the solution, applying two- and three-component analysis. Reasonably, the quantitative data obtained by the two- and by three-component approach are in close agreement.

It is frustrating to conclude that the calculated energy differences of the studied tautomeric species agree only qualitatively with the experimental data. Inclusion of the solvent effect as polarizable continuum medium improves the results significantly, but not enough considering the stability of the diketo tautomer (c). On the other hand, the optimized geometries and the vertical excitation energies are in accordance with the experiment. The complicated mechanism of the studied tautomerism is possibly the reason for the poor agreement between the theoretical models and the experimental data. As mentioned above, the tautomerism is accompanied with rotation about the C-C bonds. That is why the solvent molecules play crucial role in the mechanism and the dynamics of the studied tautomeric processes.

## Competing interests

The authors declare that there are no competing interests.

## Authors’ contributions

AA performed all calculations and the steady-state spectroscopic measurements. SS and VK performed the synthesis, purification and structural verification of the compound. LA generated the main idea of the research and performed the spectral decomposition analysis and discussion of the flash-photolysis data. AA and LA wrote and finalized the manuscript. All co-authors edited and agreed with the present form of the manuscript. All authors read and approved the final manuscript.

## Supplementary Material

Additional file 1: Table S1 Solvent effect on the absorption maxima of compound 1. Click here for file

Additional file 2: Table S2 K_T_ values and the corresponding ΔG. Click here for file

Additional file 3: Figure S3 Graphical presentation of the temperature dependence of the integral area of the subbands p3-p7. **Table S3**. Complete list of the data obtained from the decomposition procedure (A_max_, ν_1/2_, λ_max_) for all 7 components, p1-p7, of the experimental spectra recorded in ethanol at different temperatures, 20-60°C, and the calculated individual area, I_i_. Click here for file

Additional file 4 Comparison of the calculated energy differences between the possible isomers of compound 1 and their dipole moment as obtained from different methods of calculations. Click here for file
