# Supplementary material for: Tautomerism of 4,4′-dihydroxy-1,1′-naphthaldazine studied by experimental and theoretical methods
Source: Chem Cent J. 2013 Feb 11;7:29. doi: 10.1186/1752-153X-7-29 (PMC3599304; doi:10.1186/1752-153X-7-29)
Supplement: Additional file 1: Table S1 — Solvent effect on the absorption maxima of compound 1. [file 1752-153X-7-29-S1.doc]

Supplementary Material S1.

Table S1. Solvent effect on the absorption maxima of compound **1**.

| Solvent | Apparent maximum [nm] | Maxima determined by derivative spectroscopy [nm] |
| --- | --- | --- |
| Acetonitrile | 377 | 332, 366, 385, 406, 350* |
| Acetone | 382 | 330, 368, 387, 409, 352* |
| Dioxane | 373 | 333, 369, 388, 409, 351* |
| Diethyl ether | 382 | 333, 366, 385, 405, 350* |
| Dichloromethane | 373 | 332, 365, 383, 405, 350* |
| DMSO | 393 | 335, 377, 397, 420, 360* |
| Ethylacetate | 372 | 332, 368, 386, 408, 350* |
| Methanol | 384 | 332, 368, 386, 408, 350* |
| THF | 386 | 332, 371, 390, 412, 351* |
| Ethanol | 386 | 333, 370, 389, 410, 353* |

* not always well detectable
