# Supplementary material for: Tautomerism of 4,4′-dihydroxy-1,1′-naphthaldazine studied by experimental and theoretical methods
Source: Chem Cent J. 2013 Feb 11;7:29. doi: 10.1186/1752-153X-7-29 (PMC3599304; doi:10.1186/1752-153X-7-29)
Supplement: Additional file 2: Table S2 — KT values and the corresponding ΔG. [file 1752-153X-7-29-S2.doc]

Supplementary material S2

The K_T_ values, K_T_=[B]/[A], were estimated as the ratio between the molar fractions of two species, A and B absorbing at 386 and 330 nm, respectively. The latter were calculated from the relative decrease of the intensity of the absorption maximum at 386 nm upon temperature increase.

Table S2. K_T_ values and the corresponding ΔG

| Temperature  [K] | K_T_ | ΔG  [kcal/mol] |
| --- | --- | --- |
| 293 | 0 |  |
| 303 | 0.0234 | 2.258 |
| 313 | 0.0464 | 1.908 |
| 323 | 0.0721 | 1.686 |
| 333 | 0.0986 | 1.532 |

The obtained data were used to calculate the ΔH and ΔS values:

ΔH= 9.482 kcal/mol;

ΔS = - 24.013 cal/mol.K
