# Supplementary material for: Tautomerism of 4,4′-dihydroxy-1,1′-naphthaldazine studied by experimental and theoretical methods
Source: Chem Cent J. 2013 Feb 11;7:29. doi: 10.1186/1752-153X-7-29 (PMC3599304; doi:10.1186/1752-153X-7-29)
Supplement: Additional file 3: Figure S3 — Graphical presentation of the temperature dependence of the integral area of the subbands p3-p7. Table S3. Complete list of the data obtained from the decomposition procedure (Amax, ν1/2, λmax) for all 7 components, p1-p7, of the experimental spectra recorded in ethanol at different temperatures, 20-60°C, and the calculated individual area, Ii. [file 1752-153X-7-29-S3.doc]

Supplementary Material S3.

Spectral decomposition into subbands and 3D fitting of the data for obtaining the molar fraction of each species

The temperature dependent spectra in ethanol were decomposed into 7 components and the area of each component was calculated. The overall data are summarized in Table S3 and depicted in Fig.S3. The position of the peaks was estimated by derivative spectroscopy as well and is given in the legend in Fig.S3 for the sake of comparison with the data obtained from the decomposition procedure, listed in Table S3.

**Fig.S3**. Graphical presentation of the temperature dependence of the integral area of the subbands p3-p7.

The subbands p3, p5 and p6 are attributed to the diol form, **a**, and the sum of the corresponding area is denoted as $I_{diol}^{0}$. The peak p4 is attributed to the diketo form, **c**, and the p7 subband – to the monoketo form, **b**. This assignment was taken into account in the 3D fitting of the data and the in estimation of the molar fraction of each species, described further on.

Table S3. Complete list of the data obtained from the decomposition procedure (A_max_, ν_1/2_, λ_max_) for all 7 components, p1-p7, of the experimental spectra recorded in ethanol at different temperatures, 20-60^0^C, and the calculated individual area, I_i_.

| T/p_i_ | A_max_ | ν_1/2_ | λ_max_ | I_i_ |
| --- | --- | --- | --- | --- |
| **20 ^0^C** |  |  |  |  |
| p1 | 0.776 | 4694.066 | 213.452 | 3872.07871 |
| p2 | 0.744 | 4419.51 | 244.447 | 3495.26671 |
| p3 | 0.145 | 4267.69 | 354.237 | 657.8004 |
| p4 | 0.115 | 8498.58 | 324.527 | 1038.90891 |
| p5 | 0.42 | 3140.404 | 380.602 | 1402.06477 |
| p6 | 0.167 | 1606.397 | 410.418 | 285.1692 |
| p7 | 0.096 | 1221.531 | 391.519 | 124.6548 |
|  |  |  |  |  |
| 30 ^0^C |  |  |  |  |
| p1 | 0.76 | 4669.677 | 213.385 | 3772.53865 |
| p2 | 0.736 | 4435.577 | 244.248 | 3470.25351 |
| p3 | 0.136 | 4200.488 | 352.47 | 607.25615 |
| p4 | 0.115 | 8635.098 | 324.939 | 1055.59756 |
| p5 | 0.415 | 3152.191 | 379.744 | 1390.5733 |
| p6 | 0.16 | 1613.649 | 410.376 | 274.44942 |
| p7 | 0.1 | 1309.962 | 391.486 | 139.24896 |
|  |  |  |  |  |
| 40 ^0^C |  |  |  |  |
| p1 | 0.728 | 4613.817 | 213.39 | 3570.46688 |
| p2 | 0.727 | 4452.426 | 244.037 | 3440.83927 |
| p3 | 0.122 | 4029.285 | 349.558 | 522.54185 |
| p4 | 0.117 | 8927.151 | 326.092 | 1110.2787 |
| p5 | 0.406 | 3124.539 | 378.033 | 1348.48229 |
| p6 | 0.157 | 1615.209 | 410.703 | 269.56385 |
| p7 | 0.121 | 1472.281 | 391.744 | 189.3692 |
|  |  |  |  |  |
| 50 ^0^C |  |  |  |  |
| p1 | 0.682 | 4523.726 | 213.475 | 3279.54754 |
| p2 | 0.717 | 4465.735 | 243.848 | 3403.65371 |
| p3 | 0.109 | 3856.216 | 346.224 | 446.80818 |
| p4 | 0.117 | 9058.094 | 326.041 | 1126.56421 |
| p5 | 0.402 | 3143.596 | 376.48 | 1343.3403 |
| p6 | 0.141 | 1593.226 | 411.395 | 238.79749 |
| p7 | 0.141 | 1631.937 | 392.309 | 244.59961 |
|  |  |  |  |  |
| 60 ^0^C |  |  |  |  |
| p1 | 0.634 | 4429.006 | 213.555 | 2984.89316 |
| p2 | 0.706 | 4477.457 | 243.642 | 3360.23297 |
| p3 | 0.106 | 3783.438 | 344.521 | 426.31023 |
| p4 | 0.117 | 9182.119 | 325.726 | 1141.98932 |
| p5 | 0.386 | 3113.531 | 374.903 | 1277.53781 |
| p6 | 0.126 | 1595.332 | 411.933 | 213.67558 |
| p7 | 0.164 | 1787.664 | 392.694 | 311.64704 |

3D Fitting of equation (1) in order to obtain the individual area of each species, $I_{i}^{0}$, X axis is I(diOH), Y - I(monoOH), Z - I(diCO), X is I(diOH), Y - I(monoOH), Z - I(diCO).

$\frac{I_{diketo}^{i}}{I_{diketo}^{0}}+\frac{I_{monoketo}^{i}}{I_{monoketo}^{0}}+\frac{I_{diol}^{i}}{I_{diol}^{0}}=1$ (1)

Values calculated from fitting equation (1):

$I_{diol}^{0}$ = 4381.44 (p3+p5+p6)

$I_{diketo}^{0}$ = 2407.53 (p4)

$I_{monoketo}^{0}$ = 3561.44 (p7)

The calculated molar fractions and the corresponding equilibrium constants are given and discussed in the main text
