# Supplementary material for: Tautomerism of 4,4′-dihydroxy-1,1′-naphthaldazine studied by experimental and theoretical methods
Source: Chem Cent J. 2013 Feb 11;7:29. doi: 10.1186/1752-153X-7-29 (PMC3599304; doi:10.1186/1752-153X-7-29)
Supplement: Additional file 4 — Comparison of the calculated energy differences between the possible isomers of compound 1 and their dipole moment as obtained from different methods of calculations. [file 1752-153X-7-29-S4.doc]

Supplementary Material S4.

**Comparison of the calculated energy differences between the possible isomers of compound 1 and their dipole moment as obtained from different methods of calculations**

**Table 1**. Calculated (M06-2X/def2TZVP) relative energies (in kcal/mol), corrected for ZPVE, and dipole moments for the most stable conformers of the possible tautomeric forms of compound **1**.

| Tautomer | Relative energies  [kcal/mol] | Dipole moment  [Debye] |
| --- | --- | --- |
| **a-R1** | **0.00** | 0.00 |
| **a-R2** | 0.27 | 2.40 |
| **a-R3** | 0.47 | 0.29 |
| **b-R1** | 8.38 | 9.11 |
| **b-R2** | **7.94** | 9.05 |
| **b-R2’** | 8.55 | 8.32 |
| **b-R3** | 8.17 | 8.38 |
| **c** | **24.00** | 1.11 |

**Table 2**. Calculated (B3LYP/def2TZVP) relative energies (in kcal/mol), corrected for ZPVE, and dipole moments for the most stable conformers of the possible tautomeric forms of compound **1**.

| Tautomer | Relative energies  [kcal/mol] | Dipole moment  [Debye] |
| --- | --- | --- |
| **a-R1** | **0.00** | 0.00 |
| **a-R2** | 0.32 | 2.40 |
| **a-R3** | 0.59 | 0.00 |
| **b-R1** | 6.05 | 9.88 |
| **b-R2** | **5.23** | 9.61 |
| **b-R2’** | 6.40 | 9.05 |
| **b-R3** | 5.56 | 9.01 |
| **c** | **19.87** | 0.57 |

**Table 3**. Calculated (HF/def2TZVP) relative energies (in kcal/mol), corrected for ZPVE, and dipole moments for the most stable conformers of the possible tautomeric forms of compound **1**.

| Tautomer | Relative energies  [kcal/mol] | Dipole moment  [Debye] |
| --- | --- | --- |
| **a-R1** | **0.00** | 0.00 |
| **a-R2** | 0.27 | 2.38 |
| **a-R3** | 0.50 | 0.89 |
| **b-R1** | 8.65 | 9.14 |
| **b-R2** | **8.00** | 9.20 |
| **b-R2’** | 8.67 | 8.38 |
| **b-R3** | 8.05 | 8.52 |
| **c** | **19.17** | 0.79 |

**Table 4**. Calculated (HF/6-31G**) relative energies (in kcal/mol), corrected for ZPVE, and dipole moments for the most stable conformers of the possible tautomeric forms of compound **1**.

| Tautomer | Relative energies  [kcal/mol] | Dipole moment  [Debye] |
| --- | --- | --- |
| **a-R1** | **0.00** | 0.00 |
| **a-R2** | 0.29 | 2.47 |
| **a-R3** | 0.54 | 0.79 |
| **b-R1** | 9.56 | 8.74 |
| **b-R2** | **9.07** | 8.89 |
| **b-R2’** | 9.45 | 8.06 |
| **b-R3** | **8.97** | 8.27 |
| **c** | **20.10** | 0.80 |
